# The efficacy and safety of epirubicin and cyclophosphamide combined with pyrotinib in neoadjuvant treatment for HER2-positive breast cancer: A real-world study

**DOI:** 10.3389/fonc.2023.1041111

**Published:** 2023-01-30

**Authors:** Fu Li, Yimin Liang, Ming Luo, Jiayue Shen, Taosheng Zhou, Yajing Liang, Xiaoqi Tang, Huiming Yuan, Jian Zeng

**Affiliations:** Department of Gastrointestinal and Gland Surgery, The First Affiliated Hospital of Guangxi Medical University, Nanning, GuangXi, China

**Keywords:** human epidermal growth factor receptor 2 positive, breast cancer, neoadjuvant therapy, pyrotinib, real-world study

## Abstract

**Purpose:**

Long-term survival benefit of anthracyclines for human epidermal growth factor receptor 2 (HER2)-positive breast cancer is clear. In the neoadjuvant treatment, compared with the monoclonal antibody such as trastuzumab and pertuzumab, the clinical benefit of pyrotinib, a new small-molecule tyrosine kinase inhibitor (TKI), as the main anti-HER2 strategy currently requires more research to determine. Our real-world study is the first prospective observational study in China to evaluate the efficacy and safety of epirubicin (E) and cyclophosphamide (C) with pyrotinib as anti-HER2 therapy in the neoadjuvant setting of patients with stage II-III HER2-positive breast cancer.

**Methods:**

From May 2019 to December 2021, 44 untreated patients with HER2-positive nonspecific invasive breast cancer who received 4 cycles of neoadjuvant EC with pyrotinib. The primary endpoint was pathological complete response (pCR) rate. Secondary endpoints included the overall clinical response, breast pathological complete response rate (bpCR), the rate of axillary lymph nodes pathological negativity and adverse events (AEs). Other objective indicators were the rate of surgical breast-conserving, the negative conversion ratios of tumor markers.

**Results:**

Thirty-seven (84.1%) of 44 patients completed this neoadjuvant therapy, and 35 (79.5%) had surgery and were included in the primary endpoint assessment. The objective response rate (ORR) of 37 patients was 97.3%. Two patients reached clinical complete response, 34 obtained clinical partial response, 1 sustained stable disease, and no one had progressive disease. Eleven (31.4%) of 35 patients who had surgery achieved bpCR and the rate of axillary lymph nodes pathological negativity was 61.3%. The tpCR rate was 28.6% (95% CI: 12.8-44.3%). Safety was evaluated in all 44 patients. Thirty-nine (88.6%) had diarrhea, and 2 developed grade 3 diarrhea. Four (9.1%) patients had grade 4 leukopenia. All grade 3-4 AEs could be improved after symptomatic treatment.

**Conclusion:**

The regimen of 4 cycles of EC combined with pyrotinib presented some feasibility in the neoadjuvant setting for HER2-positive breast cancer with manageable safety. New regimens with pyrotinib should be evaluated for higher pCR in future.

**Trial registration:**

chictr.org Identifier: ChiCTR1900026061.

## 1 Introduction

Breast cancer has become the most common malignant tumor in women (1). Over 20% of breast cancers are human epidermal growth factor receptor 2 (HER2) overexpressing subtypes closely related to more aggressive phenotype and poorer prognosis (2–4). The preoperative or neoadjuvant systemic treatment represents the standard approach for women with HER2-positive breast cancer at stage II or III, to downstage tumor, allow breast conserving surgery, and avoid axillary dissection (5). The achievement of pathological complete response (pCR) after neoadjuvant treatment is associated significantly with the improved survival outcomes, particularly in HER2-positive and hormone-receptor (HR) negative breast cancer patients (6).

Epirubicin (E) and cyclophosphamide (C) have been widely used in the neoadjuvant setting for HER2-positive breast cancer and a meta-analysis of 8 studies with 5354 available patients confirmed that anthracycline-based adjuvant chemotherapy would add more benefit in disease-free survival (DFS) and overall survival (OS) than non–anthracycline-based adjuvant chemotherapy for HER2-positive breast cancer (7). And there are many options for anti-HER2 therapy, such as monoclonal antibodies (mAb) and tyrosine kinase inhibitors (TKIs). TKIs including lapatinib, neratinib, tucatinib and pyrotinib can exert or enhance the anti-tumor activity on the basis of traditional chemotherapy with or without monoclonal antibody. They have all proven to be effective in reducing recurrence and prolonging survival for advanced breast cancer (8–10). Based on the phase III ExteNET study, only neratinib has been approved for the extended adjuvant treatment of patients with early-stage HER2-positive breast cancer, to follow adjuvant trastuzumab-based therapy (11). However, the indications of other TKIs need to be further explored in more neoadjuvant clinical practice.

Pyrotinib, a newly-developed oral TKI from China in 2018, can irreversibly inhibit HER1, HER2, and HER4 (12). In the phase III PHOBE trial, progression-free survival (PFS) was significantly higher in the pyrotinib plus capecitabine arm than that in the lapatinib plus capecitabine arm for patients with advanced HER2-positive breast cancer (12.5 months vs 6.8 months, hazard ratio 0.39, *p* < 0.0001) (9). Moreover, some randomized controlled studies of neoadjuvant therapy for HER2-positive breast cancer have shown that the clinical effect of lapatinib is not superior to that of trastuzumab (13–16). The target of pyrotinib is comprehensive and irreversible relative to lapatinib. Therefore, our real-world study is the first clinical observational study in China to evaluate the efficacy and safety of epirubicin and cyclophosphamide (EC) with pyrotinib as the main anti-HER2 therapy in neoadjuvant therapy for patients with HER2-positive breast cancer.

## 2 Methods

### 2.1 Patients and study design

This prospective observational study was designed to evaluate the efficacy and safety of EC combined with pyrotinib as neoadjuvant therapy for patients with HER2-positive breast cancer. Inclusion criteria were as follows: patients who have been fully informed and have provided written informed consent; untreated female patients with unilateral or bilateral biopsy diagnosed invasive breast cancer with stage II/III; aged ≥18 years; the result of HER2 status was 3+ by immunohistochemistry (IHC) or 2+ but positive amplification by fluorescence *in situ* hybridization (FISH); with one or more measurable lesions by breast ultrasound, mammography or magnetic resonance imaging (MRI). Exclusion criteria: pregnant or recent intention to conceive; inflammatory breast cancer or distant metastasis; considered ineligible by a physician. All patients received necessary medical imaging examinations to confirm clinical staging before neoadjuvant therapy and surgery, including ultrasonography, chest and abdomen computed tomography (CT), bone scan, and breast MRI if necessary. Patients who received less than a full cycle of pyrotinib or allocated to other targeted therapy due to disease progression were excluded in the efficacy evaluation in our study.

This real-world study included patients with HER2-positive nonspecific invasive breast cancer who were seen at The First Affiliated Hospital of Guangxi Medical University from May 2019 to December 2021 and received neoadjuvant therapy with 4 cycles of EC plus pyrotinib. Clinical data will be collected from medical records, including demographic data, imaging examinations and laboratory testing. Our study has been registered with the Chinese Clinical Trials Registry (ChiCTR1900026061).

### 2.2 Treatments and procedures

Epirubicin (100mg/m^2^) and cyclophosphamide (600mg/m^2^) intravenously were given every 3 weeks for 4 cycles with oral pyrotinib at an initial dose of 400 mg/day, 21 days as a cycle for 4 consecutive cycles until further progression, unacceptable toxicity, or medical decision. We administrated the results of bone marrow, renal and hepatic laboratory examination on the 3rd, 6th, and 9th days of each subsequent cycle to ensure adequate function. After completing 4 cycles of neoadjuvant therapy and preoperative examinations, patients received individualized breast surgery, including mastectomy or breast-conserving surgery, with or without breast reconstruction. Sentinel lymph node biopsy (SLNB) is the standard method for patients with clinically negative axillary lymph nodes after neoadjuvant therapy, and it is also one of the basis for further axillary lymph node dissection (ALND) (17). In this study, axillary lymph node management was performed after neoadjuvant therapy according to the China Anti-cancer Association Committee of Breast Cancer Society (CACA-CBCS) 2021 guidelines. If patients achieved total pathological complete response, the adjuvant therapy were changed to docetaxel (T) once every 3 weeks for 4 cycles combined with pyrotinib and trastuzumab (H) for 1 year. If not, patients received docetaxel for 4 cycles combined with trastuzumab and pertuzumab (P) every 3 weeks for 1 year.

### 2.3 Endpoint definition and assessments

The primary endpoint of the study was pCR rate. The pCR was defined by no invasive residual cancer in the breast and no pathological involvement of axillary lymph nodes. The presence of intraductal carcinoma *in situ* (DCIS) was permitted (ypT0/is ypN0). Secondary endpoints included: clinical response assessment and objective response rate (ORR) before surgery; breast pathological complete response (bpCR) rate (defined as the absence of invasive cancer in the breast); the rate of axillary lymph nodes pathological negativity; the incidence and grade of adverse events (AEs). AEs were recorded for patients with pyrotinib for at least one full cycle. Other indicators were collected including: the rate of surgical breast-conserving, the negative conversion ratios of tumor markers. Clinical response was assessed by ultrasound, mammography, or MRI according to the Response Evaluation Criteria in Solid Tumors (RECIST) version 1.1. The -assessment of breast pathological response was based on the Miller-Payne (MP) grading system. Safety assessments were based on National Cancer Institute Common Terminology Criteria for Adverse Events (NCI CTCAE) version 5.0.

### 2.4 Statistical analyses

Patient characteristics, secondary endpoints, and incidence of AEs were summarized descriptively. Categorical variables were performed as frequency counts (percentage) and continuous variables were performed as median (range). The proportion pCR and 95% confidence interval (CI) were calculated by the Clopper-Pearson method. P values < 0.05 were considered significant. All tests were performed in a two-sided manner. All analyses were performed using the IBM SPSS statistics version 26.

## 3 Results

### 3.1 Patient characteristics

**Figure 1:**
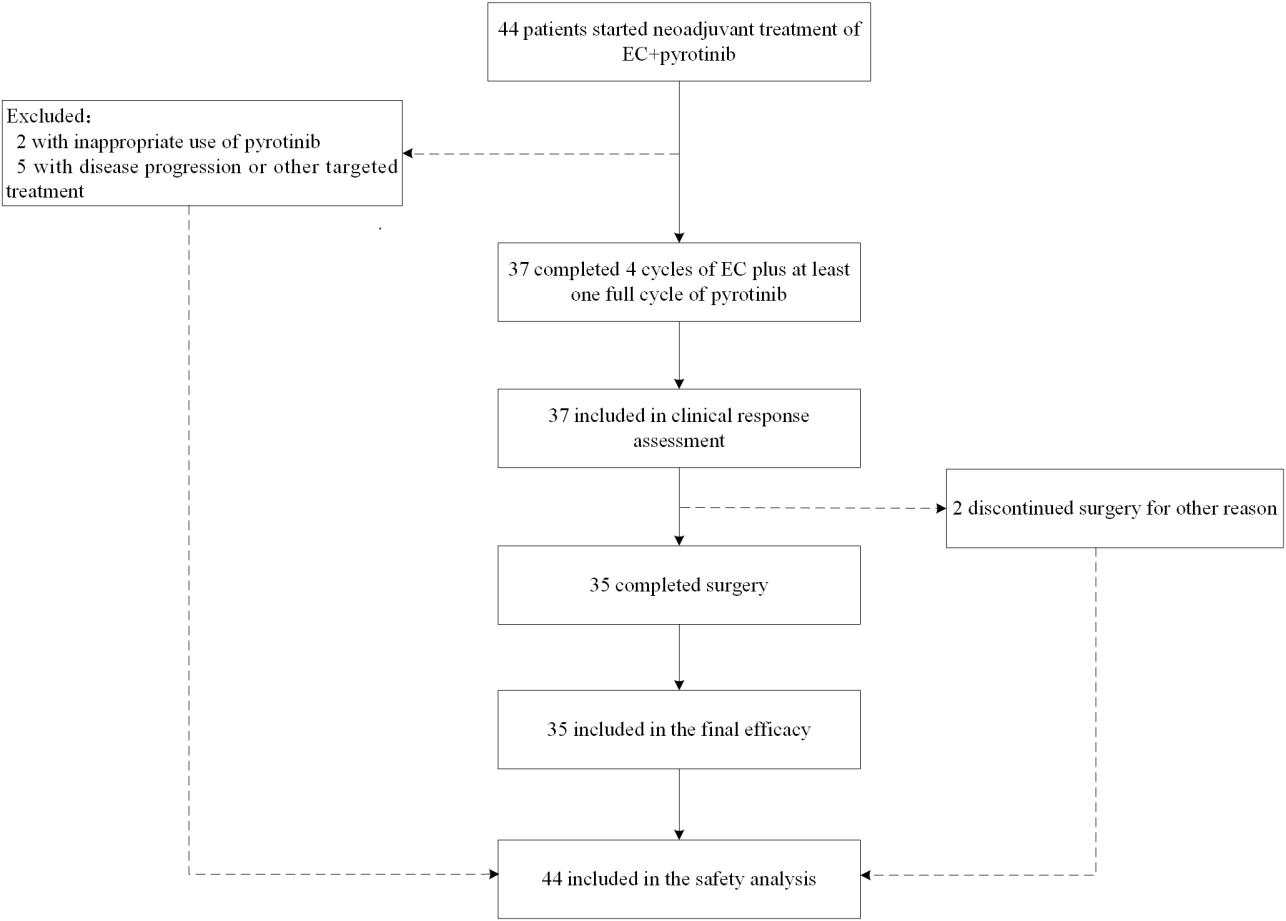
Trial profile.

From May 2019 to December 2021, 44 untreated patients with HER2-positive nonspecific invasive breast cancer who received EC with pyrotinib in neoadjuvant therapy were enrolled. All 44 patients were included in the safety analysis. Thirty-seven (84.1%) patients completed 4 cycles of EC plus pyrotinib, and were included in clinical response assessment and ORR calculation. Two patients withdrew from surgical treatment: one refused to have surgery and the other was transferred to intensive care units (ICU) for treatment-unrelated disease after neoadjuvant therapy. Thirty-five (79.5%) patients completed surgery with postoperative pathological results and were evaluated for final efficacy. **Figure 1** shows the trial profile and **Table 1** describes baseline characteristics of patient and tumor. The median age was 46.5 years (range: 22-64). Forty-two (95.5%) were considered axillary lymph node clinical positive by imagological diagnosis. However, they were not confirmed by sentinel lymph node biopsy before neoadjuvant therapy. It is worth noting that one patient diagnosed with bilateral primary invasive ductal carcinoma with histological grade 2 on the left side and grade 3 on the right side. HER2 status of both sides were 2+ by IHC, and HER2 amplification confirmed by FISH. Both sides achieved clinical partial response (PR) after neoadjuvant treatment. Finally, she was performed bilateral mastectomy.

**Table 1:** Patients characteristics at baseline.

Patients characteristics	N=44
Median age (range)	46.5 (22-64)
Menopausal status, n (%)	
Premenopausal	26 (59.1)
Postmenopausal	18 (40.9)
Histological grade, n (%)	
II	29 (65.9)
III	6 (13.6)
Unknow	9 (20.5)
Tumor size, n (%)	
≤20mm	4 (9.1)
20-50mm	26 (59.1)
>50mm	14 (31.8)
Clinical stage, n (%)	
II	25 (56.8)
III	19 (43.2)
Clinical nodal status, n (%)	
Positive	42 (95.5)
Negative	2 (4.5)
HER2 status, n (%)	
IHC 3+	34 (77.3)
IHC 2+, FISH+	10 (22.7)
ER status, n (%)	
Positive	18 (40.9)
Negative	26 (59.1)
PR status, n (%)	
Positive	12 (27.3)
Negative	32 (72.7)
HR status, n(%)	
ER and/or PR positive	19 (43.2)
ER and PR negative	25 (56.8)
Ki67, n (%)	
<20%	13 (29.5)
≥20%	31 (70.5)

### 3.2 Efficacy

Of 37 patients who completed 4 cycles EC plus pyrotinib, three (8.1%) started pyrotinib from the second and third cycle for personal reasons. **Table 2** shows the treatment period and dose reductions of pyrotinib. The median time from first medication to surgery was 91 (range:76-125) days. After neoadjuvant therapy, imaging assessment revealed no primary breast lesions in two (5.4%) patients, and clinical negative axillary lymph nodes in 17 (45.9%) patients. Two (5.4%) patients achieved CR, 34(91.9%) reached PR, one (2.7%) sustained SD, and no one had PD. The ORR rate was 97.3%. Two patients who received pyrotinib for less than 4 cycles also achieved PR. Among 35 patients who had surgery, 11 patients (31.4%, 95% CI: 15.2-47.6) achieved bpCR. Two of 35 patients had step reduction of pyrotinib due to side effects but continued the standard chemotherapy with a good pathological response: one was reduced to 320mg form the third cycle with MP4 pathological response; the other was reduced to 320mg from the second cycle with MP3 pathological response.

**Table 2:** The period and dose reductions of pyrotinib.

Dose and cycles of pyrotinib (mg/qd)	n (%)
400×4	32 (86.5)
400×2+320×2	1 (2.7)
400×1+320×3	1 (2.7)
400×3	2 (5.4)
400×2	1 (2.7)

**Figure 2:**
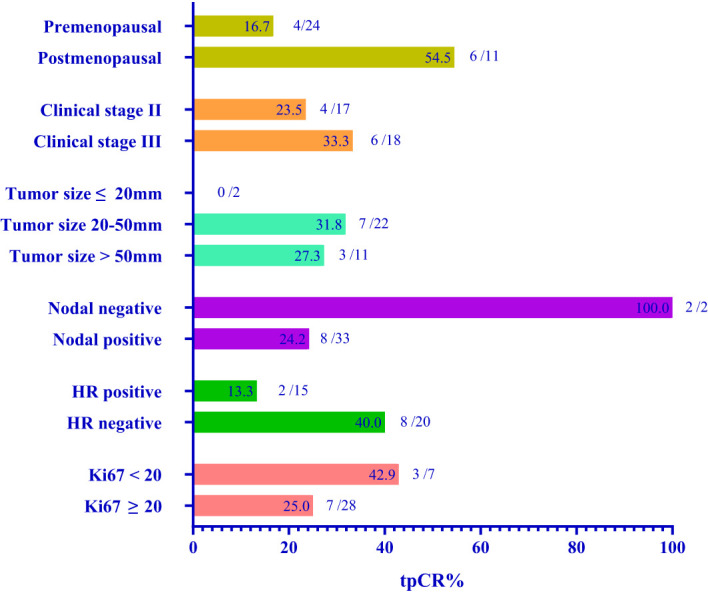
Subgroup analysis.

We routinely performed sentinel lymph node biopsy in surgery, and the surgeon decided whether to perform ALND according to the sentinel lymph node biopsy and the actual situation. Thirty-one (88.6%) of 35 patients underwent ALND, of which 19 (61.3%) of 31 patients showed no pathological axillary lymph node metastasis. The rate of axillary lymph nodes pathological negativity was 61.3% (95% CI: 43.1-79.5%). One patient achieved bpCR but had a micro-metastasis in the axillary lymph node. Therefore, 10 (28.6%) of 35 patients achieved total pathological complete response (tpCR) of breast and axillary lymph nodes, and the tpCR rate was 28.6% (95% CI, 12.8-44.3%). We performed breast-conserving surgery on 10 (28.6%) patients, of whom 1 (2.9%) patient avoided ALND due to intra-operative condition. Before and after neoadjuvant treatment, we routinely detected serum tumor markers, such as AFP, CEA, CA125, CA153 and CA199. The negative conversion ratios of CEA, CA125, and CA153, which were considered to be related to breast cancer, were 72.7%, 50.0%, and 77.8% (**Table 3**). Subgroup analysis of tpCR was performed according to menopausal status, clinical stage, tumor size, clinical lymph node status, hormone receptor status, and Ki-67, as shown in **Figure 2**.

**Table 3:** The negative conversion ratio of tumor markers after neoadjuvant therapy.

Tumor markers	Positive result before, n (%)	Positive result after, n (%)	The negative conversion ratio, n (%)
AFP	0/37	0/36*	0
CEA	11/37 (29.7)	3/36* (8.3)	8/11 (72.7)
CA125	2/37 (5.4)	1/36* (2.8)	1/2 (50)
CA153	9/37 (24.3)	1/36* (2.8)	7/9 (77.8)
CA199	0/37 (0)	1/36* (2.8)	0

* One patient lost data.

### 3.3 Safety

AEs were observed in patients who complete at least one cycle of neoadjuvant pyrotinib. All 44 patients were included in the safety assessment. Thirty-two (86.5%) of 37 patients completed the neoadjuvant cycles of pyrotinib. Two patients reduced the dose to 320mg in the second and the third cycle respectively due to side effects. Three patients started pyrotinib in the second or third cycle of chemotherapy for personal reasons without dosage reduction. No one reduced the dose of pyrotinib to 240mg. AEs and grades are shown in **Table 4**. Diarrhea was the most common AE, occurring in 39 (88.6%) patients with grade 3 diarrhea in 2 (4.5%) patients. All diarrhea relieved after symptomatic treatment. The most serious AE was grade 4 leukopenia, which occurred in 4 (9.1%) patients even though after routine use of prophylactic granulocyte colony-stimulating factor (G-CSF). In addition, six patients developed grade 1-2 vomiting, and two had grade 1-2 fever. Leukopenia, nausea, vomiting, and fever were mainly considered to be caused by EC. No one withdrew from treatment because of intolerable side effects.

**Table 4:** Adverse events.

Adverse event N = 44	All grades, n (%)	Grade 1 n (%)	Grade, n (%)	Grade 3 n (%)	Grade 4 n (%)
Diarrhea	39 (88.6)	30 (68.2)	7 (15.9)	2 (4.5)	0
Leukopenia	20 (45.5)	3 (6.8)	6 (13.6)	7 (15.9)	4 (9.1)
Nausea	2 (4.6)	1 (2.3)	1 (2.3)	0	0
Vomiting	6 (13.6)	3 (5.7)	3 (6.8)	0	0
dizzy	1 (2.3)	1 (2.3)	0	0	0
Fever	2 (4.6)	1 (2.3)	1 (2.3)	0	0

## 4 Discussion

HER2 is a member of the human epidermal growth factor receptor (EGFR) family and controlling proliferation, survival, and apoptosis of cells (18). Overexpression and/or amplification of HER2 were expressed in approximately 20% breast cancer patients, which was related to more aggressive biological behaviors and poorer clinical outcomes (4). The anti-HER2 therapy has become the essential approach for patients with HER2-positive breast cancer. However, in the neoadjuvant treatment, compared with monoclonal antibody such as trastuzumab and pertuzumab, the clinical benefit of small-molecule TKIs as the main anti-HER2 strategy currently requires more research to determine. And there are also a variety of options for matching chemotherapy regimens. In clinical practice, AC-THP is one of the recommended neoadjuvant therapy for HER2-positive breast cancer. The anthracyclines include epirubicin, doxorubicin, pirarubicin and they are equally effective (19). For patients with early-stage HER2-positive breast cancer, 4 cycles of doxorubicin and cyclophosphamide in neoadjuvant treatment can significantly shrink the tumor size and increase the pCR rate (20). However, the cardiotoxicity of anthracyclines and monoclonal antibody are an important concern in clinical practice.

Pyrotinib (Jiangsu Hengrui Medicine) is a small-molecular irreversible dual pan-HER TKI that blocks HER1, HER2 and HER4, with the best clinical benefit at an oral dose of 400mg per day (12). A phase II clinical trial revealed that pyrotinib plus capecitabine was significantly superior to lapatinib plus capecitabine in OS and PFS for previously treated advanced breast cancer (21). Two randomized, multicenter phase III trials, PHENIX study and PHOEBE study further verified that pyrotinib combined with capecitabine has a significantly better prognosis than that of lapatinib combined with capecitabine in such patients (9, 19, 22). Two real-world studies also suggested pyrotinib to be a preferred option over lapatinib in the treatment of advanced breast cancer patients (23, 24). The clinical benefit of pyrotinib in patients with advanced breast cancer is significantly better than that of lapatinib, perhaps because pyrotinib have a wider target and irreversible inhibitory activity, and different mechanisms that can be used to overcome trastuzumab resistance (25).

Whether TKIs can be comparable to trastuzumab as a single anti-HER2 strategy remains inconclusive. In a number of previous trails on lapatinib in neoadjuvant therapy, dual-targeted regimen of trastuzumab and lapatinib can significantly improve the pCR rate, while the difference between pCR rates of trastuzumab and lapatinib alone are not statistically significant (13, 15, 26). In a phase 3 head-to-head study of GeparQuinto GBG 44, patients who received EC-T were divided into 2 groups and received neoadjuvant therapy in combination with lapatinib or trastuzumab respectively. The pCR rate of trastuzumab group was significantly higher than of the lapatinib group (30.3% vs 22.7%, OR: 0.68, 95% CI: 0.47–0.97, *p* = 0.04), suggesting that lapatinib is not suitable as a single anti-HER2 therapy in combination with neoadjuvant chemotherapy (14).

In the I-SPY 2 adaptive phase II trial of neoadjuvant HER2-positive breast cancer, neratinib was administrated based on the chemotherapy of paclitaxel, and achieved an ideal pCR rate threshold in the HR-negative group (56%, 95% Bayesian probability interval [PI]: 37 to 73%), significantly higher than the trastuzumab group (33%, 95% PI: 11 to 54%) (27). In the NSABP FB-7 phase II trial, pCR rates between the trastuzumab arm (38%, 95% CI: 24–54%) and the neratinib arm (33%, 95% CI: 20–50%) were numerically similar, and it was suggested combining trastuzumab plus neratinib with paclitaxel could provide a greater pCR rate of 50% (95% CI: 34-66%), especially for HR-negative patients (28). The CTNEoBC pooled analysis has confirmed that patients with HER2-overexpressing breast cancer who received neoadjuvant HER2-targeted therapy were more likely to achieve higher pCR rates which are associated with better long-term survival (6).

In this real-world study, we aimed to evaluate the efficacy and safety of chemotherapy regimen with 4 cycles EC combined with pyrotinib as anti-HER2 strategy in neoadjuvant setting. After 4 cycles of neoadjuvant treatment, 2 of 37 patients had CR, 34 had PR, and one had SD. The ORR rate was 97.3%. The tpCR rate was 28.6% (95% CI: 12.8-44.3%). Subgroup analysis indicated that this neoadjuvant therapy maybe more suitable for estrogen receptor-negative patients with more possibility to achieve pCR. Moreover, the results of subgroup analysis showed that postmenopausal patients, patients with advanced clinical stage, and patients with Ki67<20% had higher tpCR rate. The subgroup analysis did not contain age because the sample size of patients under 35 years old (6 of 35) or over 60 years old (1 of 35) was small.

A phase II neoadjuvant clinical trial of 19 people showed that the tpCR rate of 8 cycles of EC-T combined with pyrotinib + trastuzumab dual-HER2 targeted therapy was 73.7%, which was numerically much higher than those of the KRISTINE trial (55.7%) and the BERENICE trial (60.7%) of the dual-targeted regimen of trastuzumab + pertuzumab (29–31). But its simple size was too small to draw any definitive conclusions. In the recent phase II Panphila trial using 6 cycles of neoadjuvant docetaxel + carboplatin + trastuzumab (TCH) plus pyrotinib, the preoperative ORR rate was 100%, with 38 (55.1%) of 69 patients achieving tpCR (32). The phase III PHEDRA trial confirmed the feasibility of 4 cycles of docetaxel combined with trastuzumab + pyrotinib for dual- HER2 targeted therapy with the ORR rate of 91.6%, the bpCR rate of 43.8%, and the tpCR rate of 41% (33). The lower tpCR rate in our study may be due to fewer cycles (only 4 cycles) of neoadjuvant chemotherapy with pyrotinib and the absence of enhanced anti-HER2 therapy with monoclonal antibody. The clinical stages of the patients in our study were all in stage II-III, the proportion of patients whose tumor diameter greater than 5cm by imaging assessment accounted for 31.8% and those with clinical positive lymph nodes occupied as high as 95.5%, which may be related to lower tpCR rate. At the same time, 3 patients had a desynchronized or reduced dose of pyrotinib due to personal reasons or side effects. For one patient who had SD with MP2 pathological response, the details suggested that the tumor cells had necrotic foci and it was considered that the tumor did not shrink significantly due to the necrosis of reactive tumor cells had not been absorbed after chemotherapy. It is notable that the skin symptoms of the ulcers of the patient were significantly improved, and the tumor regression may be achieved by prolonging the treatment period. There was one patient who had bilateral primary breast cancer achieving PR for both sides after neoadjuvant therapy with postoperative pathology of MP2, suggesting that neoadjuvant therapy for bilateral breast cancer should also be lengthened or be appended with endocrine, radiotherapy and other therapies after operation. In addition, our study showed promising results in tumor marker clearance and improved feasibility of breast-conserving surgery.

In clinical practice, we pursue the high rate of pCR. Now, Trastuzumab deruxtecan (T-DXd, formerly DS-8201) is a highly effective HER2-targeted antibody drug conjugate (ADC) with a topoisomerase I inhibitor payload, which is the first choice in advanced HER2-positive breast cancer for second-line treatment recommended by 2021 ESMO Clinical Practice Guideline (34) and 2022 ASCO Guideline Update (35). T-DXd has attempted to be used in neoadjuvant therapy for HER2-positve breast cancer in clinical trials, the results are worth expecting. Nevertheless, we ignore the economic factors and fail to distinguish the role of one target medicine combination chemotherapy drugs from multiple target drugs combination chemotherapy drugs. In this study, we try our best to explore the efficacy and safety of EC combined with pyrotinib in neoadjuvant treatment for HER2-positive breast cancer, which may be an economic and effective therapeutic strategy for neoadjuvant therapy of HER2 positive breast cancer.

We observed 44 patients who had started this neoadjuvant therapy and had received at least one cycle medicine of pyrotinib. The most common AE was diarrhea occurring in 39 patients (88.6%) with ​​different grades. Two (4.5%) of them had grade 3 diarrhea that could be improved by symptomatic treatment. The most serious AE was leukopenia and 4 patients experienced grade 4 leukopenia. Even though we routinely used prophylactic treatment of chemotherapy-induced leukopenia after each cycle of chemotherapy, some patients with grade 3-4 leukopenia still required re-injections to maintain normal bone marrow function, and symptomatic reduction of chemotherapy if diagnosed with myelosuppression.

The limitations of our study are small sample size and lack of controlled and randomized comparison. This study is a single-center study and there are no follow-up prognostic data to determine the long-term impact of this regimen. Further collection of survival data and randomized controlled studies with larger sample sizes are needed in the future to verify the efficacy and long-term benefit of this neoadjuvant treatment.

## Conclusions

In conclusion, this was the first real-world study to evaluate the efficacy and safety of EC combined with pyrotinib in neoadjuvant treatment for HER2-positive breast cancer. Although the various standard neoadjuvant regimens for HER2-positive breast cancer from related breast cancer guidelines are widely used in clinical practice, the results of our study provide evidence on the feasibility and safety of the TKIs combined with anthracycline and cyclophosphamide as a new neoadjuvant therapy for HER2-positive breast cancer. Our results showed a novel anti-HER2 approach for patients with HER2-positive breast cancer with relatively short medical duration, good clinical outcome and manageable adverse events. And compared with trastuzumab and pertuzumab in china, the cost is lower with considerable clinical benefit. Our findings also implied that patients with estrogen receptor-negative characteristics may be preferred for this neoadjuvant therapy. Although our study has showed some clinical benefit, new anti-HER2 drug was developed and new regimens using pyrotinib should be assessed in next trials to achieve the similar high pCR close to 60% as with TCHP.

## Data availability statement

The raw data supporting the conclusions of this article will be made available by the authors, without undue reservation.

## Ethics statement

The studies involving human participants were reviewed and approved by China Ethics Committee of Registering Clinical Trials. The patients/participants provided their written informed consent to participate in this study.

## Author contributions

FL and JZ contributed to the study conception and design. FL, YL, ML, JS, TZ, YJL, XT, HY, and JZ contributed to the acquisition of data. YL analyzed and interpreted the data. YL drafted the manuscript. FL and JZ contributed to the critical review and revision of the manuscript. All authors contributed to the article and approved the submitted version.
